# Robust Multi-Subtype Identification of Breast Cancer Pathological Images Based on a Dual-Branch Frequency Domain Fusion Network

**DOI:** 10.3390/s25010240

**Published:** 2025-01-03

**Authors:** Jianjun Li, Kaiyue Wang, Xiaozhe Jiang

**Affiliations:** School of Mechanical and Electrical Engineering, China Jiliang University, Hangzhou 310018, China

**Keywords:** frequency domain, feature fusion, histopathological classification, deep learning, breast cancer

## Abstract

Breast cancer (BC) is one of the most lethal cancers worldwide, and its early diagnosis is critical for improving patient survival rates. However, the extraction of key information from complex medical images and the attainment of high-precision classification present a significant challenge. In the field of signal processing, texture-rich images typically exhibit periodic patterns and structures, which are manifested as significant energy concentrations at specific frequencies in the frequency domain. Given the above considerations, this study is designed to explore the application of frequency domain analysis in BC histopathological classification. This study proposes the dual-branch adaptive frequency domain fusion network (AFFNet), designed to enable each branch to specialize in distinct frequency domain features of pathological images. Additionally, two different frequency domain approaches, namely Multi-Spectral Channel Attention (MSCA) and Fourier Filtering Enhancement Operator (FFEO), are employed to enhance the texture features of pathological images and minimize information loss. Moreover, the contributions of the two branches at different stages are dynamically adjusted by a frequency-domain-adaptive fusion strategy to accommodate the complexity and multi-scale features of pathological images. The experimental results, based on two public BC histopathological image datasets, corroborate the idea that AFFNet outperforms 10 state-of-the-art image classification methods, underscoring its effectiveness and superiority in this domain.

## 1. Introduction

According to a report by the World Health Organization (WHO), breast cancer (BC) is one of the most common cancers among females and a leading cause of cancer-related deaths in females under 45 years old. In 2022, there were 19.96 million new cancer cases worldwide, and BC ranked second in terms of incidence, becoming the leading cause of cancer-related deaths in 112 countries globally [1]. Early detection and accurate diagnosis of tumors play a critical role in clinical treatment, which can significantly reduce the mortality of cancers, exhibiting profound implications for treatment and prognosis among cancer patients. Therefore, early identification and appropriate treatment contribute to improving the long-term survival of patients with BC [2,3].

There are various methods for the detection and diagnosis of BC. Among them, histopathological examination is considered the “gold standard” for the diagnosis of this cancer [4]. Pathological images can present structural information at the cellular and tissue levels of breast lesions, which is critical for the diagnosis, classification, and grading of BC [5,6,7,8]. However, the intrinsic complexity and diversity of breast histopathological images result in labor-intensive and time-consuming work for pathologists. Furthermore, variations in experience and the subjective application of pathological diagnostic standards often bring inconsistent and non-reproducible diagnostic outcomes. To improve diagnostic accuracy, reduce diagnostic time, and avoid misdiagnosis or missed diagnosis among BC patients, deep learning (DL)-based computer-aided detection and diagnosis systems have received increasing attention from researchers. DL overcomes the limitations of conventional classification methods that require manual feature extraction of lesions. It can autonomously learn the characteristics of BC lesion regions, enabling it to assist clinicians in diagnosing diseases accurately and rapidly, minimizing subjective judgments and reliance on experience, and reducing the risk of misdiagnosis and missed diagnosis.

In recent years, DL algorithms have provided unique solutions for clinical medical image recognition. Spanhol et al. [9] employed various patching strategies to automatically extract features from BC histopathological images using AlexNet [10] while preserving high image resolution. Jiang et al. [11] proposed a Transformer-based [12] fine-grained classification model (Breast TransFG Plus) to perform grading of invasive ductal carcinoma (IDC). This model eliminated the widespread distribution characteristics of tumor cells in hematoxylin–eosin (H&E)-stained pathological images. Su et al. [13] introduced a model-ensemble learning framework that integrated classical Vision Transformer (ViT) modules into a graph neural network (GNN) for cancer tissue image grading. In this framework, graph nodes [14] corresponded to nucleus feature extraction based on convolutional neural networks (CNNs), while the Transformer module captured global graph node features of individual whole-slide images (WSIs). The evaluation results based on two public datasets [15,16] demonstrated that the ensemble model outperformed single models. However, these methods often focus solely on feature extraction in the spatial domain, overlooking the rich information potentially embedded in the frequency domain. The information is crucial for identifying microscopic structures and periodic texture patterns in pathological images, revealing complex changes in tumor cells and interactions between tissues. To address the aforementioned issues, we propose the following content:

- We propose a dual-branch framework, AFFNet, designed to simultaneously capture fine-grained local details and global structural information in images.
- The characteristics and attention mechanisms of BC histopathological images are analyzed from the perspective of the frequency domain. MSCA is employed to effectively extract the local high-saliency features of pathological images. Meanwhile, FFEO is utilized to enhance high-frequency information while preserving the low-frequency features of images. These frequency domain features are then transformed into spatial domain feature maps, thus increasing the number of feature maps and enabling the network to focus on contour and texture information simultaneously.
- A novel frequency-domain-adaptive feature fusion strategy is proposed to adaptively model multi-scale contextual information. This enables the model to efficiently integrate multi-source information at different resolutions, thus enhancing its representational capacity.
- AFFNet outperforms other state-of-the-art models in the experiments based on two publicly accessible BC histopathological image datasets. Furthermore, the effectiveness and superiority of the proposed method are validated by a T-SNE-based visualization.

In the following, Section 2 presents related work; Section 3 describes the proposed AFFNet model; Section 4 elucidates the extensive experimental results in detail; Section 5 provides the conclusion of this study, highlighting the advantages, limitations, and future potential of this method.

## 2. Related Work

**Frequency domain**: The pivotal role of the frequency domain in the field of DL has been increasingly recognized. Through the frequency domain, DL has demonstrated great potential in fields like image processing, signal processing, and time–series analysis [17,18,19]. In recent years, many researchers have employed frequency domain methods to enhance the feature extraction performance of models [20,21,22]. DCCNet [23] strengthens token interactions by applying dynamic circular convolutions in a separable manner along the channel dimension in the frequency domain. Specformer [24] integrates spectral analysis with multi-head self-attention into the original ViT architecture, exhibiting improved performance. Shen et al. [25] adopted branch processing techniques to obtain high, mid, and low frequencies and demonstrated the effectiveness of FFDN in extracting frequency domain information, further fully exploiting multi-band information in the shallow feature maps of blurry images.

**Multi-branch networks**: In recent years, a multi-branch approach has been adopted in an increasing number of designs to improve network structures and enhance performance. SCNet [26] divides input features into two branches. Specifically, one branch utilizes self-calibrated convolutions to significantly expand the receptive field of each convolutional layer through internal information interaction, thereby enhancing feature representation; while the other branch preserves the integrity of the original features. Wang et al. [27] proposed a dual-path lesion-aware neural network to remove the challenges of high inter-class similarity and significant intra-class variability in colonoscopy images through a dual-network fusion strategy, thereby improving the discriminative ability of the network. Jiang et al. [28] developed an end-to-end dual-branch fusion network to tackle the challenges of recognizing small details and complex nodules in lung nodule segmentation, significantly improving the segmentation performance for hard-to-identify lung nodules. These studies validate that multi-branch structural designs can effectively improve the performance of DL models on specific tasks.

## 3. Proposed Method

**Figure 1:**
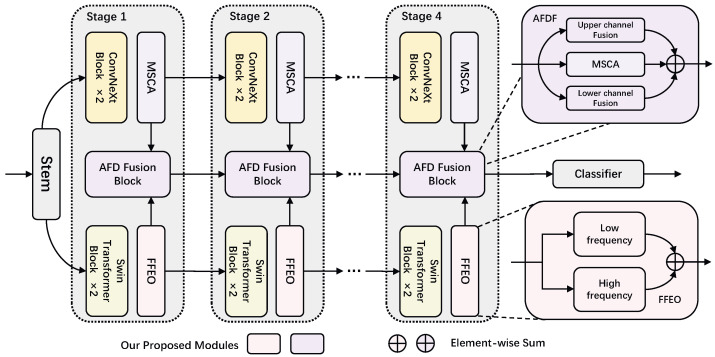
The hierarchical network architecture of AFFNet.

This study proposes a novel medical image analysis framework to assist in the histopathological diagnosis of BC. The architecture of AFFNet is illustrated in Figure 1. This network is established by ingeniously integrating the strengths of ConvNeXt [29] and Swin Transformer [30] through various frequency domain methods. It can not only capture detailed local features of breast tissues but also extract critical global contextual information. The design principles and functional implementation of each component in the network will be elucidated in the following sections.

### 3.1. Stem

**Figure 2:**
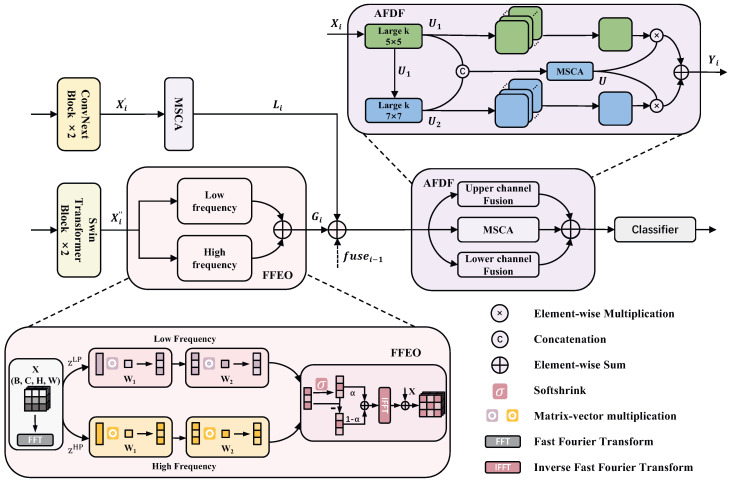
The basic architecture in each stage of AFFNet.

In the AFFNet architecture, the image is first passed through a shared stem layer, where downsampling is performed via convolutions to reduce the input image resolution to one-fourth of its original size. Subsequently, the downsampled image is distributed to two specifically designed feature encoding branches. AFFNet builds hierarchical feature maps across four stages, with each stage’s architecture illustrated in Figure 2. The figure also offers a detailed depiction of two key modules: the Fourier Filtering Enhancement Operator (FFEO) and Adaptive Frequency Domain Fusion (AFDF).

### 3.2. Frequency Domain Operator

#### 3.2.1. Multi-Spectral Channel Attention

The challenges in classifying histopathological images of BC include the nuclear overlap (which affects the features of individual nuclei) and the potential presence of multiple types of nuclei (which results in the indistinct features of some nuclei), thereby significantly affecting classification performance. Therefore, effectively extracting and utilizing salient features becomes a key to improving classification accuracy. To more effectively capture and leverage key frequency domain components in the signals, we introduced MSCA. This module employs the discrete cosine transform (DCT) to amplify essential information and suppress irrelevant details, followed by channel compression and re-excitation operations to enhance classification accuracy.

For an image *f* with a size of *H × W*, its discrete cosine transform can be expressed as follows:

(1) $$\begin{array}{cc} \; & F\left(u,v\right)=\sum_{h=0}^{H-1}\sum_{w=0}^{W-1}f\left(h,w\right)\varphi \left(u,v,h,w\right)\\ \; & s.t.\;h\in \{0,1,\cdots ,H-1\},w\in \{0,1,\cdots ,W-1\} \end{array}$$

Here, *h* and *w* are spatial domain indices representing the rows and columns of the image pixels. *f*(*h*,*w*) denotes the pixel value at (*h*,*w)* in the spatial domain, while *H* and *W* are the image dimensions in the vertical and horizontal directions, respectively. φ(·) represents the basis function for the 2D DCT. *u* and *v* are frequency domain indices corresponding to the vertical and horizontal directions of the image, and *F*(*u*,*v*) represents the spectral information obtained after the 2D DCT.

First, the input $X\in R^{C\times H\times W}$ is divided along the channel dimension into *n* segments $X^{i}\in R^{\frac{C}{n}\times H\times W}$ (where *C* must be divisible by *n*; in this study, n=16). After division, *X* is represented as $\left[X^{0},X^{1},X^{2},\cdots ,X^{n-1}\right]$, and the corresponding frequency components are assigned to $X^{i}$. Consequently, the 2D DCT spectrum of the entire input *X* is expressed as:

(2) $$Freq=Concat(DCT\left(X^{0}\right),DCT\left(X^{1}\right),\ldots ,DCT\left(X^{n-1}\right))$$

Next, *Freq* undergoes the excitation and rescale steps:

(3) Y=MSCA(X)=Rescale(Excitation(Freq))

The result obtained from Equation (3) represents the output *Y* of the MSCA module. By incorporating frequency domain information, the original input undergoes a transformation, enhancing its texture features from low to high significance and thereby improving the original features at the texture level.

#### 3.2.2. Fourier Filtering Enhancement Operator

In histopathological image analysis, high-frequency information encapsulates critical microstructural details, which play a pivotal role in improving the classification performance and robustness of the model. In conventional methods, ideal low-pass filters are used to suppress high-frequency noise while preserving the low-frequency features of images. However, the high-frequency details that are crucial for pathological diagnosis may be overlooked, which is detrimental to the accurate diagnosis and classification of pathological images. To overcome this problem, the FFEO is proposed in this study to enhance high-frequency information while retaining low-frequency features of images. By performing precise modulation in the frequency domain, FFEO identifies and retains high-frequency details valuable for pathological analysis while reducing irrelevant noise. The Algorithm 1 section provides a detailed description of the pseudo-algorithm process of FFEO.
**Algorithm 1:** Fourier Filtering Enhancement Operator**Input: **$X_{\mathrm{in}}=X\in R^{C\times H\times W}$**Output: **$X_{\mathrm{out}}=Y\in R^{C\times H\times W}$1:$B,C,H,W\leftarrow \mathrm{shape}\;\mathrm{of}\;X_{\mathrm{in}}$  2:$z\leftarrow \mathrm{RFFT}2(X_{\mathrm{in}}.\;\mathrm{permute}).\;\mathrm{reshape}\left(B,H,W//2+1,N,C/N\right)$    **Filtering:**  3:${\tilde{z}}_{u,v}^{LP},{\tilde{z}}_{u,v}^{HP}\leftarrow \mathrm{Calculate}\;\mathrm{of}\;\mathrm{low}-\;\mathrm{and}\;\mathrm{high}-\mathrm{frequency}\;\mathrm{components}\;\mathrm{by}\;\mathrm{Equations}\;(4)\;\mathrm{and}\;(5)$  4:${\tilde{z}}_{u,v}=\left[{\tilde{z}}_{u,v}^{LP},{\tilde{z}}_{u,v}^{HP}\right]$    **Enhancement:**  5:${\tilde{z}}_{u,v}^{\prime }=\alpha \times \mathrm{softshrink}\left({\tilde{z}}_{u,v}\right)+\left(1-\alpha \right)\times \left({\tilde{z}}_{u,v}-\mathrm{softshrink}\left({\tilde{z}}_{u,v}\right)\right)$  6:${X^{\prime }}_{in}\leftarrow \mathrm{IRFFT}2\left({\tilde{z}}^{\prime }\right).\;\mathrm{reshape}\left(B,C,H,W\right)$  7:$X_{\mathrm{out}}\leftarrow Y=X_{\mathrm{in}}^{\prime }+X$  
8:**return** $X_{\mathrm{out}}$

**Introduction**: The Fourier transform simultaneously captures both the low-frequency and high-frequency components of a signal. To retain low-frequency information while enhancing high-frequency components, we apply the Fourier transform to convert the input *X* from the spatial domain to the frequency domain.

**Fourier filter design**: First, the input $X\in R^{C\times H\times W}$ is transformed into frequency domain features *z* using the Fourier transform, where each component is represented as $z_{u,v}$, with *u* and *v* as frequency domain indices. Next, a two-layer perceptron (MLP) is applied to the frequency domain features derived from the Fourier Transform, allowing the network to capture complex representations across multiple levels. As shown in Equations (4), (5), (6), the MLP separately models high- and low-frequency features, facilitating comprehensive analysis and processing of the entire frequency spectrum.

(4) $${\tilde{z}}_{u,v}^{LP}=\mathrm{MLP}\left(z_{u,v}^{LP}\right)=W_{2}\sigma \left(W_{1}z_{u,v}^{LP}+b_{1}\right)+b_{2}\;$$

(5) $${\tilde{z}}_{u,v}^{HP}=\mathrm{MLP}\left(z_{u,v}^{HP}\right)=W_{2}\sigma \left(W_{1}z_{u,v}^{HP}+b_{1}\right)+b_{2}\;$$

(6) $${\tilde{z}}_{u,v}=\left[{\tilde{z}}_{u,v}^{LP},{\tilde{z}}_{u,v}^{HP}\right]$$

Inspired by a previous study [31], the weights are structured in a block-diagonal format, with each *W* comprising *N* distinct small weight blocks. Each small weight block is shared among all $z_{u,v}$. The variables $z_{u,v}^{LP}$ and $z_{u,v}^{HP}$ represent the low-pass and high-pass components, where $z_{u,v}^{LP}\in C^{low\_freq\_count}$, $z_{u,v}^{HP}\in C^{high\_freq\_count}$, $low\_freq\_count=high\_freq\_count=\frac{1}{2}\left(H\times \left(\frac{W}{2}+1\right)\right)$.

**Design enhancement**: In conventional methods, soft-thresholding and shrinkage in the frequency domain are used to remove insignificant high-frequency noise. However, such methods may inadvertently filter out high-frequency details that are critical for pathological diagnosis, such as cell boundaries and subtle structures of nuclei. To eliminate these limitations, the following filtering enhancement method is designed in this study.

(7) $${\tilde{z}}_{u,v}^{\prime }=\alpha \times \mathrm{softshrink}\left({\tilde{z}}_{u,v}\right)+\left(1-\alpha \right)\times \left({\tilde{z}}_{u,v}-\mathrm{softshrink}\left({\tilde{z}}_{u,v}\right)\right)$$

where α is set to 0.5. Finally, the transformation is switched back from the frequency domain to the time domain and connected with the original input *X* via a residual connection, resulting in the final output of FFEO:

(8) $$Y=FFEO\left(X\right)+X=IFFT\left({\tilde{z}}_{u,v}^{\prime }\right)+X$$

#### 3.2.3. Structure

In the histopathological image classification of BC, to achieve accurate recognition of subtle features within images, a CNN–Transformer framework is adopted, incorporating MSCA and FFEO, as described in Equations (9) and (10), which enhances the ability to capture frequency domain features of pathological images.

(9) $$L_{i}=\sigma \left(MSCA\left(X_{i}^{\prime }\right)\right)⊙X_{i}^{\prime },\left(i=1,2,3,4\right)$$

(10) $$G_{i}=\sigma \left(FFEO\left(X_{i}^{\prime \prime }\right)\right)⊙X_{i}^{\prime \prime },\left(i=1,2,3,4\right)$$

In the ConvNeXt branch, an MSCA module is embedded into each block as described in Equation (9). $X_{i}^{\prime }$ represents the output features of the i-th stage ConvNeXt block, while $L_{i}$ denotes the enhanced features of $X_{i}^{\prime }$ after processing through MSCA. The DCT effectively concentrates an image’s energy into low-frequency components, making it ideal for extracting global structural features in pathology images, such as cell distribution and tissue morphology. Integrating the MSCA module into the ConvNeXt branch enables precise suppression of non-essential information while simultaneously enhancing focus on global salient features and extracting fine local details.

In the Swin Transformer branch, an FFEO module is embedded into each block as described in Equation (10). $X_{i}^{\prime \prime }$ represents the output features of the i-th stage Swin Transformer block, while $G_{i}$ denotes the enhanced features of $X_{i}^{\prime \prime }$ after processing through FFEO. This operator employs the Fourier Transform to convert spatial domain information into frequency domain representations and dynamically enhances features within specific frequency bands. Unlike the DCT, which focuses primarily on low-frequency components, Fourier Transform captures both low- and high-frequency information, offering flexibility to emphasize high-frequency features such as cell boundaries and intra-nuclear texture details. This enhancement mechanism, combined with the Swin Transformer, enables the model to maintain global perceptual awareness while enhancing sensitivity to local fine-grained features.

### 3.3. Adaptive Frequency Domain Fusion

The extensive contextual information required for different cancer types depends on the characteristics of the lesion type and tissue structures. For instance, distinguishing breast lumps may require relatively less contextual information, whereas identifying tubular adenomas in some pathological images may demand a broader range of contextual information. Therefore, inspired by a previous study [32], multi-scale feature maps are constructed to adaptively model long-term contexts, thus assisting the model in identifying and locating objects at the different resolution. This is achieved by explicitly decomposing a large-kernel convolution into a sequence of depthwise convolutions at larger scales to form a series of large-kernel convolutions across different scales. First, the input $X_{i}$ for the current stage is constructed by combining the current stage’s local features $L_{i}$, global features $G_{i}$, and the fused output $fuse_{i-1}$ from the previous stage, where *i* denotes the stage number in the network architecture, $fuse_{0}=0$.

(11) $$X_{i}=L_{i}+G_{i}+fuse_{i-1},\left(i=1,2,3,4\right)$$

To enable $X_{i}$ to capture multi-scale spatial information with varying kernel sizes, depthwise separable convolutions of different kernel sizes are applied. The formulas for $U_{1}$ and $U_{2}$ at each layer are given as follows:

(12) $$U_{1}={\mathrm{DP}}^{5\times 5}\left(X_{i}\right),U_{2}={\mathrm{DP}}^{7\times 7}\left(U_{1}\right)$$

where DP(·) represents the depthwise separable convolution operation. The adaptive frequency domain fusion module is divided into three branches, with the middle branch and the upper and lower branches derived from Equations (13) and (14):

(13) $$U=MSCA(cat\left(U_{1},U_{2}\right))$$

(14) $$Y_{up}^{b,1,h,w}=\sum_{i=1}^{c}U_{1}^{b,c,h,w},Y_{down}^{b,1,h,w}=\sum_{i=1}^{c}U_{2}^{b,c,h,w}$$

Finally, the middle feature branch is fused with the upper and lower weight branches using Equation (15):

(15) $$Y_{i}=U\times Y_{up}^{b,1,h,w}+U\times Y_{down}^{b,1,h,w},(i=1,2,3,4)$$

## 4. Experiments and Results

### 4.1. Dataset

The model is trained and evaluated based on the data from two different BC histopathological datasets.

**BreaKHis** [33]: The experiment is conducted based on the BreaKHis dataset, which is divided into four magnifications (40×, 100×, 200×, and 400×) and includes 7909 images of eight different BC subtypes. The categories comprise benign lesions, including adenosis (A), fibroadenoma (F), phyllodes tumor (PT), and tubular adenoma (TA), as well as malignant lesions, such as ductal carcinoma (DC), lobular carcinoma (LC), mucinous carcinoma (MC), and papillary carcinoma (PC). The original image size is 700 × 460 pixels, which is resized to 224 × 224 pixels for the experiments. The dataset is divided into the training, validation, and test sets at a ratio of 0.6:0.2:0.2.

**BACH** [34]: This is a public dataset from the Grand Challenge, comprising 400 H&E-stained histopathological BC images with a size of 2048 × 1536 pixels. It can be categorized into four classes: normal, benign, in situ, and invasive. Due to the large image size and limited dataset, the following efforts are made to prevent overfitting during training. (1) Each image (2048 × 1536 pixels) is divided into smaller patches with a size of 512 × 512 pixels. (2) The patches with nuclei less than 20 are excluded as per a previous study [35]. (3) The images are resized to 224 × 224 pixels. The processed dataset contains a total of 4707 images, with 2825 for training, 941 for validation, and 941 for testing.

### 4.2. Experimental Setup

To evaluate the performance of the model, Accuracy, Precision, Recall, and F1-score are selected as classification metrics. The definition of these evaluation metrics can be expressed as follows:

(16) $$Accuracy=\frac{TP+TN}{TP+TN+FP+FN}$$

(17) $$Precision=\frac{TP}{TP+FP}$$

(18) $$Recall=\frac{TP}{TP+FN}$$

(19) $$F1=2\times \frac{Precision\times Recall}{Precision+Recall}=\frac{2TP}{2TP+FP+FN}$$

The loss is calculated using the categorical cross-entropy loss function:

(20) $$CrossEntropyLoss=-\frac{1}{N}\sum_{i=1}^{N}\sum_{c=1}^{M}y_{ic}\log{\hat{y}}_{ic}$$

where *N* represents the total number of samples; *M* represents the number of classes. $y_{ic}$ represents the target label, which takes the value 1 if the true class of sample *i* equals *c*, and 0 otherwise. ${\hat{y}}_{ic}$ represents the probability that the model predicts that sample *i* belongs to class *c*.

All experiments are conducted on a single NVIDIA GeForce RTX 3090 GPU with 24 GB of memory. The Adam optimizer is used to train the model in the training process. The initial learning rate is set to 1 ×$10^{-4}$, the batch size to 16, and the number of epochs to 100. The learning rate is adjusted using a cosine annealing schedule. A weight decay parameter of 0.01 is employed to prevent overfitting of the model. The performance of the model is optimized by tuning these parameters, thus ensuring greater robustness in the training process.

### 4.3. Results

To validate the effectiveness of the proposed model, AFFNet is compared with 10 state-of-the-art network models developed in recent years, including ConvNeXt, Swin Transformer, ViT, DFFormer [36], TransNeXt [37], CSwin [38], PoolFormer [39], BiFormer [40], StoHisNet [41], and CAS-ViT [42].

#### 4.3.1. Results Based on the BreaKHis Dataset

Table 1, Table 2, Table 3 and Table 4 present the experimental results based on the BreaKHis dataset under a magnification of 40×, 100×, 200×, and 400× across different networks. The experimental results demonstrate that although the dataset contains various typical and atypical BC subtypes, the proposed algorithm exhibits enhanced BC classification performance, and it outperforms existing methods across all four magnification levels.

Table 1 lists the performance of different comparison methods under a magnification of 40× based on the BreaKHis dataset. It can be observed that the proposed method outperforms other competitors in terms of accuracy, precision, recall, and F1-score. In the experiment based on the test dataset, AFFNet performs well, with the accuracy, precision, recall, and F1-score being 93.00%, 92.75%, 91.69%, and 92.06%, respectively. Compared with ConvNeXt and Swin Transformer, the accuracy of AFFNet increases by 16% and 7.5%, respectively. Among other methods, ViT has the lowest accuracy, which may be attributed to the insufficient number of samples in the dataset. The proposed method also shows advantages compared with stronger baselines such as TransNeXt, BiFormer, and CSwin. The results indicate that the features of images can be captured more effectively by integrating the proposed modules, contributing to improved performance.

Table 1 also lists the total number of parameters and floating-point operations (FLOPs) for this method compared with the aforementioned models. In this study, the depth of the original baseline model is modified, a hierarchical structure of [2, 2, 2, 2] is adopted to fuse the two baseline models, and frequency domain operators are also introduced, thus improving model performance while reducing computational complexity. The FLOPs of AFFNet are only 9.24 G, with 68.45 M parameters, representing a reduction of 6.11 G and 5.92 G in FLOPs and 19.12 M and 18.3 M in parameters compared with ConNeXt and Transformer, respectively. The model structure and computational efficiency of AFFNet are optimized while maintaining high accuracy, making it a more efficient and practical option for real-world applications.

Table 2, Table 3 and Table 4 present the classification results based on the BreaKHis dataset under a magnification of 100×, 200×, and 400×, respectively. It can be found that the proposed model consistently achieves optimal performance across all three magnifications. Under a magnification of 100×, ViT has the lowest accuracy. The reason is that ViT cannot construct hierarchical feature maps in the processing of images, and it directly divides the image into patches and processes them through a Transformer encoder. This approach may alter the original spatial locations of cancer regions, preventing accurate identification of cancer types. The relatively low accuracy of ConvNeXt may be attributed to the significant differences in features and structures between pathological and natural images. Pathological images often contain finer textures and patterns, while ConvNeXt, originally designed for natural images, may fail to capture these subtle features adequately. This limitation results in its suboptimal performance in medical image processing tasks. Additionally, under a magnification of 100×, the accuracy of all models except ConvNeXt and ViT reaches above 80%, with StoHisNet up to 88.70%. This model also employs a dual-branch hybrid architecture, enabling effective extraction of image features for the accurate classification of most cancer types. Similarly, under a magnification of 400×, TransNeXt outperforms other models with the accuracy reaching 89.01%. Its multi-scale reasoning capability effectively avoids deep degradation, thus maintaining efficient classification performance across images of varying magnifications.

As magnification increases, metrics such as accuracy and sensitivity exhibit a declining trend, which is closely associated with the substantial reduction in the field of view. With a reduced field of view, the type and richness of information differ significantly between low-magnification and high-magnification images. At low magnifications, the image reveals a more complete organizational structure and contextual information, with these global features aiding the model in capturing macro-level pathological features, thereby improving classification performance. However, at high magnifications, while the image contains more detailed features, the absence of global contextual support makes it challenging for local features to accurately represent the overall pathological condition, thereby weakening the model’s discriminative ability. Additionally, high-magnification images often exhibit higher noise levels, including uneven staining, artifacts, and increased texture complexity. These noise factors can interfere with the model’s feature extraction process at high magnifications, impairing its ability to recognize key pathological features. Furthermore, the greater variability in cell morphology and distribution in high-magnification images increases data heterogeneity, further complicating the classification task. This phenomenon does not indicate model failure, but rather reflects the natural variation in the characteristics of pathological images at different magnifications. It is important to emphasize that, despite the performance decline at higher magnifications, our model still outperforms all comparison models across all metrics. This suggests that our model has significant advantages in feature extraction and learning capabilities, enabling effective adaptation to and analysis of images across various magnification levels.

#### 4.3.2. Results on Based the BACH Dataset

To further explore the generalization capability of AFFNet, some experiments are also performed based on the BACH dataset (see Table 5).

In the experiment based on the BreaKHis dataset, Swin Transformer demonstrates superior performance compared with ConvNeXt, with the average accuracy increased by 5.33%. However, in the experiment based on the BACH dataset, ConvNeXt significantly outperforms Swin Transformer. This indicates that different models may perform variably based on different datasets. However, AFFNet, by integrating the strengths of both ConvNeXt and Swin Transformer architectures, demonstrates optimal performance on all datasets, highlighting its consistency and robustness compared to other models. The model fusion effectively mitigates the differences in feature distributions across datasets, compensating for the limitations of individual models in certain scenarios. This fusion strategy enhances the model’s adaptability to diverse features, enabling it to exhibit stronger performance across various datasets, thus offering a reliable solution for breast lesion classification.

#### 4.3.3. Result Analysis

**Figure 3:**
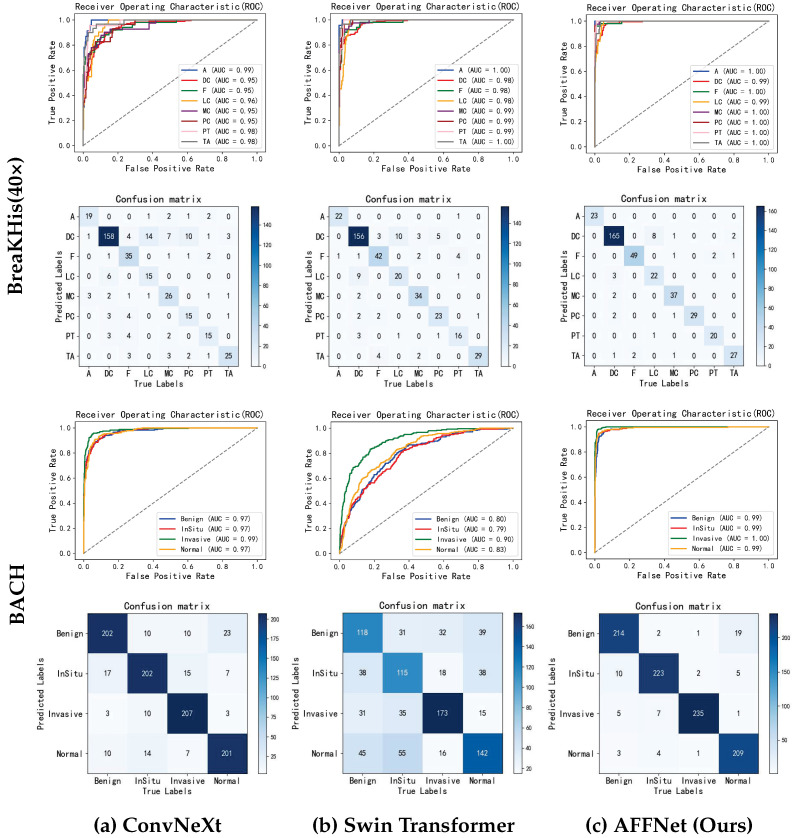
ROC and confusion matrix.

The ROC curves and confusion matrices for the baseline models and AFFNet are shown in Figure 3. The errors are analyzed based on the classification results. In the BreaKHis dataset, the classification results for DC and LC are unfavorable, while the classification of other classes is more accurate. The main reasons are elucidated as follows. Firstly, the images in the dataset are scaled, resulting in changes in their resolution. Secondly, some pathological images have unclear background information, which interferes with the judgment of the model. Thirdly, DC and LC are both invasive subtypes of BC with similar cell arrangement patterns, making it difficult for the model to make differentiation during feature extraction. In the BACH dataset, the classification results for benign and in situ are unfavorable. The main reason is that processing high-resolution images by dividing them into smaller patches may result in the loss of critical pathological features and contextual information. However, overall, the proposed model significantly outperforms the two baseline models.

**Figure 4:**
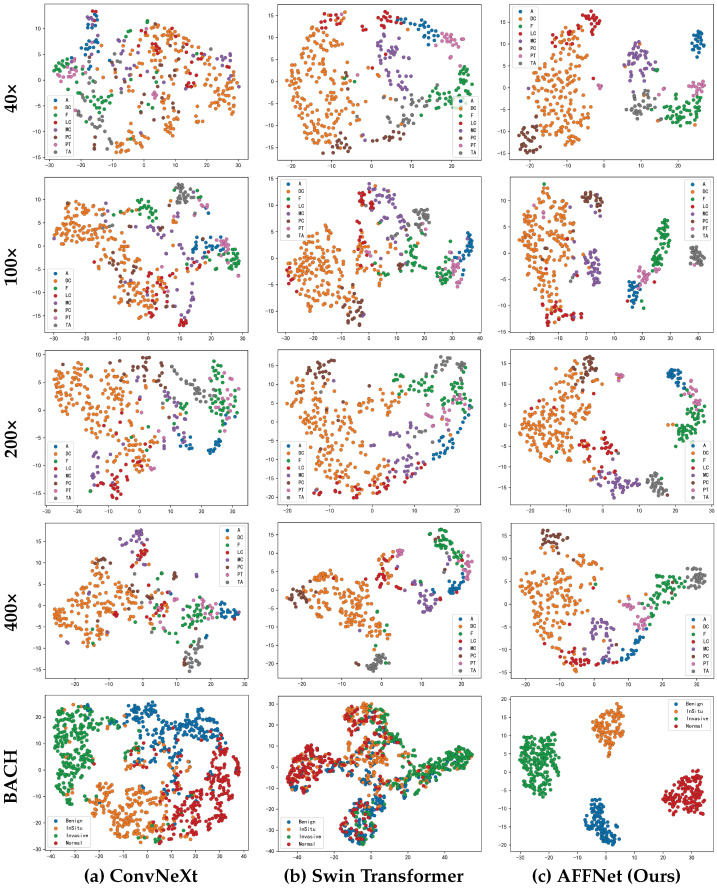
Visualization of T-SNE of different networks.

The classification ability of AFFNet is significantly superior to the two baseline networks before hierarchical fusion, which can also be confirmed by the T-SNE visualization in Figure 4. Figure 4a,b illustrate the visualizations of ConvNeXt and Swin Transformer, respectively. As shown in the figures, the inter-class distances are small, the boundaries within the classes are blurry, and the clustering effect is poor. Figure 4c shows the network proposed in this study. Obviously, this network has distinctly separated the different classes. This further confirms that this network achieves better classification performance compared with the two baseline networks in Figure 4a,b.

### 4.4. Ablation Study

To address the task of multi-subtype classification of breast pathology images, we developed a dual-branch network leveraging frequency domain fusion. This section systematically evaluates the effectiveness of each key module and strategy by adding or modifying specific components of the model. Ablation studies were conducted to address two primary questions: (1) the contribution of each component to the network architecture, and (2) the impact of the fusion strategy.

To evaluate the effectiveness of each component in our model, we utilized 40× magnification data from the BreakHis dataset for ablation experiments. These experiments enabled us to quantify and analyze the specific contribution of each component to the overall model performance. The results are presented in Table 6.

In this study, ConvNeXt and Swin Transformer were chosen as baseline models, denoted as Baseline 1 and Baseline 2, respectively. On the breast pathology image multi-subtype classification task, Baseline 1 achieved an accuracy of 77.00%, and Baseline 2 achieved 85.75%. To investigate the potential of model fusion, we performed the Baseline 1 + Baseline 2 experiment (third row) by employing a simple addition method to fuse the two baseline models. This approach enabled a direct comparison of performance differences between individual models and the fused model. Experimental results demonstrated that the fused network achieved an accuracy of 88.50%, improving performance by 11.50% and 2.75% over Baseline 1 and Baseline 2, respectively. In the Baseline 1 + MSCA experiment (fourth row), the introduction of MSCA enhanced network performance by 12.75%. In the Baseline 2 + FFEO experiment (fifth row), incorporating the FFEO improved the model performance by 5.25%. Finally, an adaptive frequency domain fusion strategy was applied to fuse the enhanced Baseline 1 and Baseline 2 at multiple scales (sixth row). This multi-scale frequency domain fusion method significantly enhanced model performance. Compared to the baseline models, our AFFNet achieved the best results on the breast pathology image multi-subtype classification task, demonstrating substantial performance improvements.

This series of ablation experiments validated the effectiveness of each network component and emphasized the critical role of the fusion strategy in enhancing model performance. Specifically, MSCA and FFEO significantly improved the model’s ability to capture critical features, while the multi-scale frequency domain fusion strategy further enhanced classification accuracy. These findings provide robust experimental evidence supporting the effectiveness of our model design.

## 5. Discussion

In this study, a dual-branch adaptive frequency domain fusion network (AFFNet) is developed for the histopathological image classification of breast cancer. This study emphasizes the importance of the frequency domain in the classification of histopathological images of breast cancer. In addition, Multi-Spectral Channel Attention is employed in the local feature branch to enhance the representation of local salient features in pathological images. The Fourier Filtering Enhancement Operator is proposed to assist the global feature branch in enhancing high-frequency information while preserving low-frequency features and reducing the interference of irrelevant noise. On that basis, the feature maps of different scales are constructed to adaptively model contextual information for both branches. Moreover, AFFNet is comprehensively evaluated based on the BreaKHis and BACH datasets. The results demonstrate that it outperforms existing state-of-the-art methods. Considering the specificity of pathological images, the performance of this network may be future improved by image enhancement techniques. Furthermore, we plan to explore the potential applications of AFFNet in other medical imaging domains, thus promoting its broader applicability in medical image processing. The findings of this study are expected to provide strong support and innovative insights for various downstream tasks in medical image analysis.

## Figures and Tables

**Table 1:** Experimental results under a magnification of 40×. (The best results are in bold.)

Model	Params (M)	Folps (G)	Accuracy (%)	Precision (%)	Recall (%)	F1-Score (%)
ConvNeXt-B	87.57	15.35	77.00	73.78	69.70	71.05
Swin Transformer-B	86.75	15.16	85.50	83.76	83.04	83.27
ViT	85.80	16.86	51.75	24.23	26.77	24.22
DFFormer-B36	111.94	22.10	79.75	76.99	74.49	75.51
TransNeXt-base	88.96	17.82	85.00	83.08	83.07	82.71
CSwin-B	76.62	14.37	83.50	81.51	79.64	80.19
PoolFormer-M36	55.41	8.76	81.75	80.14	80.73	80.12
BiFormer-B	56.04	9.36	90.25	89.67	89.62	89.44
StoHisNet	31.13	7.81	91.00	91.55	86.61	88.70
CAS-ViT	2.76	0.55	82.25	81.24	74.88	76.68
AFFNet (Ours)	68.45	9.24	**93.00**	**92.75**	**91.69**	**92.06**

**Table 2:** Experimental results under a magnification of 100×. (The best results are in bold.)

Model	Accuracy (%)	Precision (%)	Recall (%)	F1-Score (%)
ConvNeXt-B	79.56	76.16	74.08	74.79
Swin Transformer-B	85.81	84.59	82.13	83.09
ViT	56.97	43.21	33.26	32.25
DFFormer-B36	81.97	78.22	78.14	77.93
TransNeXt-base	87.98	85.63	86.01	85.78
CSwin-B	80.52	78.27	73.67	75.65
PoolFormer-M36	82.21	79.03	76.75	77.56
BiFormer-B	83.89	80.13	81.66	80.40
StoHisNet	88.70	87.32	85.60	86.34
CAS-ViT	83.65	81.21	77.88	79.34
AFFNet (Ours)	**90.62**	**90.82**	**87.29**	**88.74**

**Table 3:** Experimental results under a magnification of 200×. (The best results are in bold.)

Model	Accuracy (%)	Precision (%)	Recall (%)	F1-Score (%)
ConvNeXt-B	80.64	77.75	76.96	77.24
Swin Transformer-B	83.37	79.33	80.27	79.41
ViT	60.54	51.06	36.56	35.88
DFFormer-B36	83.37	80.15	80.12	79.82
TransNeXt-base	86.84	84.95	84.92	84.83
CSwin-B	83.37	78.51	76.49	77.18
PoolFormer-M36	82.38	82.20	77.00	79.05
BiFormer-B	87.84	86.85	88.34	87.26
StoHisNet	87.09	86.23	85.59	85.71
CAS-ViT	81.88	80.04	75.07	76.86
AFFNet (Ours)	**90.32**	**88.89**	**89.44**	**89.09**

**Table 4:** Experimental results under a magnification of 400×. (The best results are in bold.)

Model	Accuracy (%)	Precision (%)	Recall (%)	F1-Score (%)
ConvNeXt-B	84.89	84.20	81.77	82.28
Swin Transformer-B	88.73	86.29	84.91	85.33
ViT	60.71	58.08	39.81	41.06
DFFormer-B36	82.96	78.93	80.49	79.49
TransNeXt-base	89.01	87.29	87.05	86.98
CSwin-B	74.17	71.57	65.84	68.00
PoolFormer-M36	84.06	81.66	80.93	80.67
BiFormer-B	87.91	84.80	86.94	85.56
StoHisNet	85.71	82.84	83.33	82.89
CAS-ViT	83.51	81.43	77.58	78.90
AFFNet (Ours)	**90.65**	**90.39**	**88.16**	**88.76**

**Table 5:** Experimental results based on the BACH dataset. (The best results are in bold.)

Model	Accuracy (%)	Precision (%)	Recall (%)	F1-Score (%)
ConvNeXt-B	86.29	86.43	86.29	86.31
Swin Transformer-B	58.23	57.95	58.16	57.95
ViT	42.61	45.38	42.43	40.56
DFFormer-B36	85.54	85.55	85.51	85.50
TransNeXt-base	91.17	91.17	91.15	91.16
CSwin-B	48.88	51.17	48.88	49.18
PoolFormer-M36	83.84	83.95	83.83	83.83
BiFormer-B	84.48	84.45	84.45	84.44
StoHisNet	86.07	86.24	86.07	86.11
CAS-ViT	83.52	83.59	83.51	83.49
AFFNet (Ours)	**93.62**	**93.66**	**93.59**	**93.58**

**Table 6:** The ablation experiment results under a magnification of 40× based on the BreaKHis dataset.

Baseline 1	Baseline 2	MSCA	FFEO	AFDF	Accuracy (%)	Precision (%)	Recall (%)	F1-Score (%)
**✓**	×	×	×	×	77.00	73.78	69.70	71.05
×	**✓**	×	×	×	85.75	83.34	84.85	83.88
**✓**	**✓**	×	×	×	88.50	86.28	88.38	87.09
**✓**	×	**✓**	×	×	89.75	88.41	86.96	87.31
×	**✓**	×	**✓**	×	91.00	90.22	89.35	89.58
**✓**	**✓**	**✓**	**✓**	**✓**	93.00	92.75	91.69	92.06

## Data Availability

The original datasets in the study are available. BreaKHis: https://web.inf.ufpr.br/vri/databases/breast-cancer-histopathological-database-breakhis/ (accessed on 16 April 2023), BACH: https://iciar2018-challenge.grand-challenge.org/Dataset/ (accessed on 18 April 2023).
